# Genomic Changes Associated with Reproductive and Migratory Ecotypes in Sockeye Salmon (*Oncorhynchus nerka*)

**DOI:** 10.1093/gbe/evx215

**Published:** 2017-10-13

**Authors:** Andrew J. Veale, Michael A. Russello

**Affiliations:** 1 Department of Biology, The University of British Columbia, Kelowna, British Columbia, Canada; 2 Present address: Department of Environmental and Animal Sciences, Unitec, 139 Carrington Rd, Auckland, New Zealand

**Keywords:** life history evolution, adaptation, natural selection, single nucleotide polymorphism, RADseq, kokanee

## Abstract

Mechanisms underlying adaptive evolution can best be explored using paired populations displaying similar phenotypic divergence, illuminating the genomic changes associated with specific life history traits. Here, we used paired migratory [anadromous vs. resident (kokanee)] and reproductive [shore- vs. stream-spawning] ecotypes of sockeye salmon (*Oncorhynchus nerka*) sampled from seven lakes and two rivers spanning three catchments (Columbia, Fraser, and Skeena) in British Columbia, Canada to investigate the patterns and processes underlying their divergence. Restriction-site associated DNA sequencing was used to genotype this sampling at 7,347 single nucleotide polymorphisms, 334 of which were identified as outlier loci and candidates for divergent selection within at least one ecotype comparison. Sixty-eight of these outliers were present in two or more comparisons, with 33 detected across multiple catchments. Of particular note, one locus was detected as the most significant outlier between shore and stream-spawning ecotypes in multiple comparisons and across catchments (Columbia, Fraser, and Snake). We also detected several genomic islands of divergence, some shared among comparisons, potentially showing linked signals of differential selection. The single nucleotide polymorphisms and genomic regions identified in our study offer a range of mechanistic hypotheses associated with the genetic basis of *O. nerka* life history variation and provide novel tools for informing fisheries management.

## Introduction

Understanding the mechanisms that facilitate local adaptation and identifying the loci underlying adaptive population divergence are important goals of evolutionary biology (Nielsen 2005; Nosil and Schluter 2011). The study of parallel phenotypic evolution, the repeated emergence of similar phenotypes linked to specific habitats, can provide important insights into these evolutionary processes (Arendt and Reznick 2008; Martin and Orgogozo 2013).

Parallel phenotypic evolution may be caused by the selection of identical adaptive mutations (Nachman et al. 2003; Paaby et al. 2010); however, there are also cases where shared phenotypes arise through different mutations at a given locus (Gross et al. 2009; Chan et al. 2010) or through mutations at entirely different underlying loci (Steiner et al. 2009; Martin and Orgogozo 2013). Where the same genetic changes are associated with parallel phenotypic evolution, there are multiple potential evolutionary scenarios that can account for this pattern (Welch and Jiggins 2014). One scenario is that the same adaptive alleles arise independently in each derived population—representing true molecular convergent evolution (Arendt and Reznick 2008). Alternatively, standing variation may exist in the ancestral population that is subsequently recurrently selected when populations colonize novel habitats (Barrett and Schluter 2008). Adaptive variation could also flow between “derived” populations via the ancestral population—a situation known as the “transporter hypothesis” (Schluter and Conte 2009; Bierne et al. 2013). There are many additional scenarios that can account for patterns of shared adaptations. The classic example of a genomic study of parallel evolution comes from the evolution of multiple freshwater populations of three-spined stickleback (*Gasterosteus aculeatus*), where anciently derived alleles in a gene responsible for armor reduction (Ectodysplasin-A) have been recurrently selected in multiple freshwater populations (Colosimo 2005). This pattern has since been demonstrated across numerous other loci in stickleback (Jones et al. 2012). In this system, it appears likely that adaptive genetic variation that existed in one or more freshwater populations then spread to other freshwater populations via introgression with the marine population, although teasing apart the precise history of these events remains difficult (Welch and Jiggins 2014). Whether anciently derived, novel or introgressed from other populations, characterizing the genomic and geographical context of parallel phenotypic evolution provides vital clues about the mechanisms underlying adaptation.

Studies of the genomic bases of local adaptation have been facilitated by the advent of high-throughput genotyping methods, which allow for the identification and genotyping of thousands of genetic polymorphisms throughout the genome, enabling population genomic and association studies in nonmodel organisms (Miller et al. 2007; Baird et al. 2008; Davey et al. 2011). Studies that look to identify signals of adaptive divergence in wild populations often focus on sympatric or closely related populations that experience differential selective pressures, but are not highly diverged at neutral markers due to current or recent gene flow (Hemmer-Hansen et al. 2013; Soria-Carrasco et al. 2014; Roesti et al. 2015). This divergence with gene flow scenario has been hypothesized to generate “genomic islands of divergence” displaying high genetic differentiation, contrasting with lower differentiation across the rest of the genome (Via and West 2008; Nosil et al. 2009; Lotterhos and Whitlock 2015). Although a suite of factors may influence the distribution and size of divergent regions including genetic conflict, mutation rates, genetic drift, and chromosomal structure, divergent selection and adaptation are often implicated (Nosil et al. 2009; Feder and Nosil 2010; Bierne et al. 2011; Marques et al. 2016). Genomic islands of divergence may arise because, while adaptive differences typically occur at a small number of loci, regions surrounding them are physically linked to the beneficial alleles (Maynard-Smith and Haigh 1974; Kaplan et al. 1989). This process can lead to regions of the genome containing multiple linked divergent single nucleotide polymorphisms (SNPs) around adaptive sites, a phenomenon termed divergence hitchhiking (Via 2012). These parts of the genome that underlie reproductive isolation or adaptation may then become resistant to the homogenizing effects of gene flow, and outlier detection tests can be used to identify these divergent SNPs (Foll and Gaggiotti 2008).

Salmonids are an exemplary taxonomic group to study the genetic basis of phenotypic and life history traits because abundant environmental variation, combined with precise natal homing, creates a situation ripe for differential local adaptation (Quinn 2005). Numerous examples of phenotypic variation and local adaptation have been reported within salmonid species (Fraser et al. 2011). These findings, along with their high cultural and economic value (Schindler et al. 2010), have contributed to salmonids being the primary taxonomic group where genomic research has been effectively applied to conservation and management (Shafer et al. 2015; Garner et al. 2016). The genomic bases for migration related traits have been a specific focus in salmon, with numerous quantitative trait loci (QTL) identified (Norman et al. 2011; Hecht et al. 2012). Genome-wide association studies (GWAS) have likewise detected several regions of the genome associated with migration (Hess et al. 2016). The genomic bases for other phenotypic traits including thermal tolerance, size, and body condition have also been studied across a range of salmonids (Reid et al. 2005; Miller et al. 2012; Everett and Seeb 2014; Larson et al. 2016).

Sockeye salmon (*Oncorhynchus nerka*) exhibit tremendous life history and morphological variation, with several morphologically and ecologically divergent ecotypes linked to migratory and spawning behavior (Quinn 2005; Wood et al. 2008). All sockeye salmon spawn and spend their early life in freshwater, but while anadromous ecotypes later migrate out to sea, resident ecotypes (kokanee) remain in freshwater lakes throughout their life cycle (Groot and Margolis 1991; Quinn 2005). Sometimes these ecotypes occur sympatrically in the same lakes as genetically distinct populations (Foote et al. 1989; Taylor 2000). It remains possible to reanadromize kokanee by releasing them in rivers without access to lakes, but survival to maturation is lower by over an order of magnitude than similarly released anadromous sockeye salmon (Foerster 1947). Likewise, recently anthropogenically isolated anadromous sockeye salmon populations can survive and reproduce solely in freshwater, but these “residual” sockeye salmon too have decreased survival and do not match aspects of the physiology, growth, and morphology of kokanee (Smirnov 1959). Anadromous sockeye salmon and resident kokanee consistently exhibit a suite of heritable differences in size, morphology, early development rate, seawater adaptability, growth, and maturation that appear to be divergent adaptations that have arisen from different selective regimes associated with their anadromous versus nonanadromous life histories (Gustafson 1997). Kokanee populations are, however, polyphyletic having evolved from anadromous sockeye salmon through multiple independent postglacial freshwater colonization events (Taylor et al. 1996, 1997; Wood et al. 2008; Beacham and Withler 2017). As the “kokanee phenotype” is remarkably similar between catchments, these populations are examples of parallel evolution across a wide range of traits (Taylor et al. 1996).

Both anadromous sockeye salmon and kokanee can be further subdivided into reproductive ecotypes, with each population exhibiting a specific spawning habitat preference. These include classical “stream (or river)-spawning” ecotypes, “shore (or beach)-spawning” ecotypes that spawn on the shallow submerged shorelines of lakes or island beaches, and “black-kokanee” that also spawn on the lake benthos, but at depths down to 70 m below the lake surface (Moreira and Taylor 2015). In some lakes, multiple reproductive ecotypes co-occur, whereas in others only one may be present. For kokanee, co-occurring reproductive ecotypes are generally morphologically indistinguishable, whereas for anadromous sockeye salmon populations, some (minor) phenotypic differentiation between adjacent reproductive ecotypes can occur (Quinn 2005). Survival of sockeye salmon eggs and fry is highly variable and influenced by a wide range of environmental variables (Whitlock et al. 2015). As the spawning habitats for each reproductive ecotype differ across many of these environmental factors such as current strength, temperature fluctuation, and dissolved oxygen levels, divergent natural selection is likely to play a role underlying ecotype divergence (Taylor 2000).

Recently, there have been several studies examining the genomic bases for ecotype divergence within sockeye salmon in single, but separate systems (Lemay and Russello 2015b; Nichols et al. 2016; Larson et al. 2017). In Nichols et al. (2016), the genomic differentiation of population pairs of kokanee and anadromous sockeye salmon were evaluated from two lakes in the Snake River catchment. One of these pairs (Redfish Lake) also represented two different reproductive ecotypes (shore-spawning anadromous sockeye and stream-spawning kokanee). In Larson et al. (2017), several spawning sites representing different reproductive ecotypes of anadromous sockeye salmon (beach, river, and stream) were sampled within a single catchment in Alaska. Likewise, Lemay and Russello (2015b) investigated genomic differentiation between reproductive ecotypes (shore- vs. stream-spawning) of kokanee within Okanagan Lake, part of the Columbia River catchment. Although all three studies employed connectible restriction-site associated DNA sequencing (RADseq) methods (SbfI; Baird et al. 2008), their single-system scale largely precluded validation of the generality of these SNPs, and thus the evolutionary history of these regions of divergence remains unknown.

Here, we investigated the genomic patterns underlying ecotype divergence in sockeye salmon, employing RADseq of paired population samplings of migratory (anadromous vs. resident) and reproductive (shore- vs. stream-spawning kokanee) ecotypes sampled from seven lakes and two rivers spanning three catchments (Columbia, Fraser, and Skeena) in British Columbia, Canada. Our specific objectives were to: 1) reconstruct population genetic differentiation across a range of spatial scales including cross-drainage, among-lake and within-lake systems, including comparing our results to previously published studies; 2) identify outlier SNPs associated with ecotype divergence and investigate their generality as candidates for divergent selection; and 3) annotate surrounding genomic regions of the most predictive and divergent SNPs to inform mechanistic hypotheses related to *O. nerka* life history evolution.

## Materials and Methods

### Sampling

**Fig. 1.**
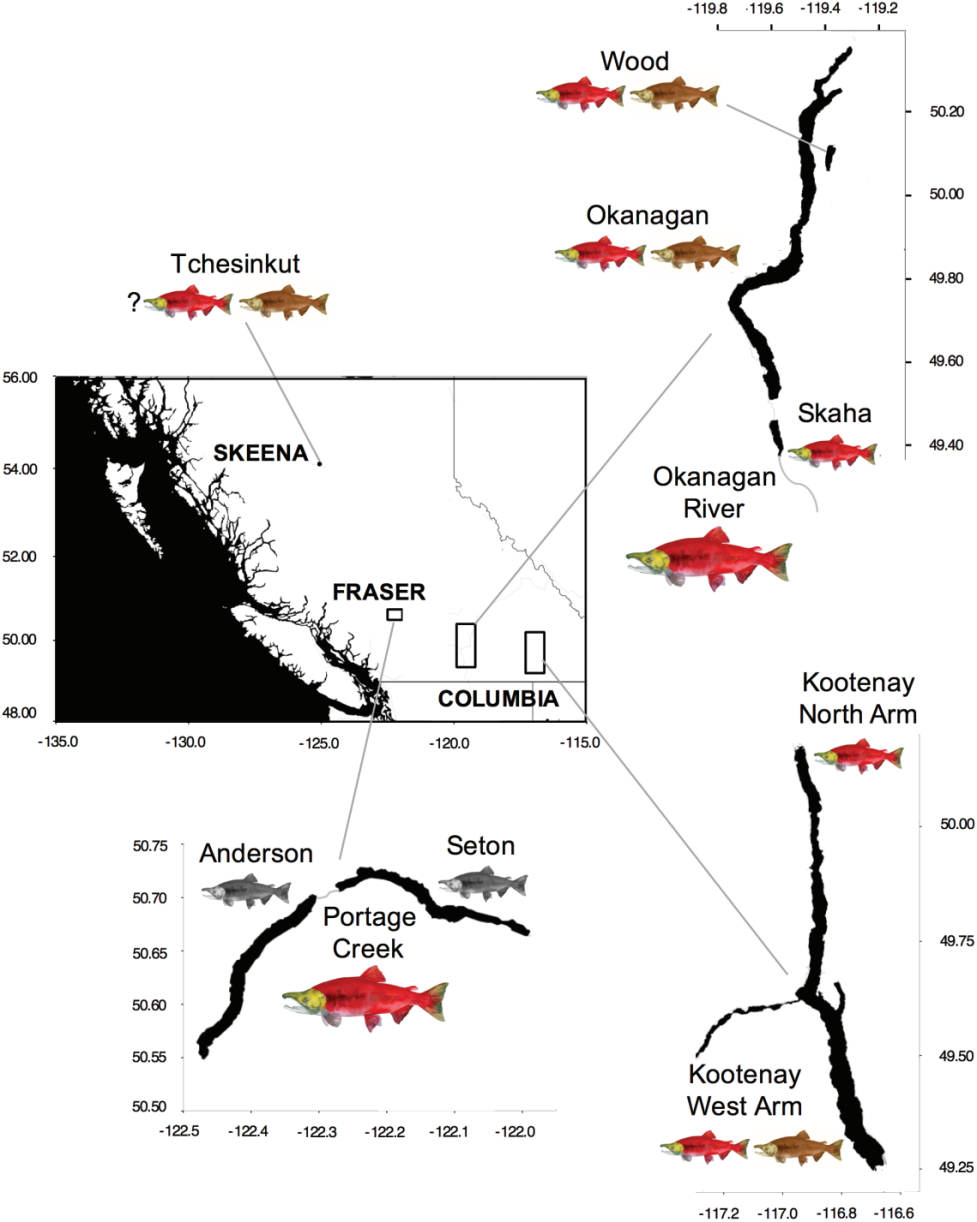
—Map of British Columbia showing the four regions where kokanee and anadromous sockeye salmon samples were obtained. The historic river catchments are indicated in the inset. Size of *Oncorhynchus nerka* images denote migratory ecotype (large = anadromous sockeye salmon, small = kokanee), colors denote reproductive ecotype (red = stream-spawning, brown = shore-spawning, gray = black kokanee). All map units in decimal degrees.

We used previously collected kokanee and anadromous sockeye salmon samples (either operculum punches or fin clips) or data from seven lakes and two rivers at the time of spawning (table 1 and fig. 1) across three historically defined catchments in BC including: 1) Columbia River (Okanagan Lake, Wood Lake, Skaha Lake, Kootenay Lake, Okanagan River); 2) Fraser River (Anderson Lake, Seton Lake, Portage Creek); and 3) Skeena River (Tchesinkut Lake). Regarding the latter, although Tchesinkut Lake is currently connected to the Fraser River drainage, there is strong evidence that kokanee populations in this region were more closely associated with the Skeena River drainage historically (Taylor et al. 1996); for the purposes of this study, we will a priori follow the reconstruction of Taylor et al. (1996). In four of the lakes (Okanagan Lake, Wood Lake, Kootenay Lake, Tchesinkut Lake), stream- and shore-spawning kokanee co-occur, and were both sampled. Because these reproductive ecotypes are morphologically indistinguishable, all samples were obtained from spawning areas during the spawning period. In Kootenay Lake, we sampled both the North Arm and West Arm populations as previous studies have demonstrated significant spatial structure (Lemay and Russello 2012; Frazer and Russello 2013). Two lake/river systems have co-occurring kokanee and anadromous sockeye salmon, including one in the Columbia River drainage (Skaha Lake kokanee—Okanagan River anadromous sockeye salmon) and one in the Fraser River drainage (Anderson Lake/Seton Lake black kokanee—Portage Creek anadromous sockeye salmon). Portage Creek links Anderson and Seton Lakes, and has previously been demonstrated to be the most closely related anadromous sockeye salmon population to these kokanee populations (Moreira and Taylor 2015). Samples from Wood, Kootenay and Tchesinkut Lakes were originally collected for Frazer and Russello (2013). Skaha Lake kokanee and Okanagan River anadromous sockeye salmon samples were provided by the British Columbia Ministry of Forests, Lands and Natural Resource Operations and previously used in Veale and Russello (2016). Samples from Portage Creek, Anderson, and Seton Lakes were originally collected for Moreira and Taylor (2015). Fully connectible data from Okanagan Lake were also used as previously collected in Lemay and Russello (2015b).

**Table 1:** *Oncorhynchus nerka* (Kokanee and Anadromous Sockeye Salmon) Samples Used in This Study, Including Migratory and Reproductive Ecotype Designations, Historical Drainage, Sample Size, Effective Population Size (*N_e_*), and Heterozygosity [Observed (*H_o_*) and Expected (*H_e_*)]

Location	Migratory Ecotype	Reproductive Ecotype	Historical Drainage	Sample Size	*N_e_* (95% CI)	*H_o_*	*H_e_*
Skaha Lake	Kokanee	Stream	Columbia River	36	619 (555–699)	0.30	0.31
Okanagan River	Anadromous	Stream	Columbia River	36	1,555 (1,347–1,839)	0.29	0.30
Okanagan Lake	Kokanee	Shore	Columbia River	48	9,851 (6,211–13,491)	0.29	0.30
	Kokanee	Stream	Columbia River	48	3,343 (2,705–4,375)	0.27	0.29
Wood Lake	Kokanee	Shore	Columbia River	36	242 (249–256)	0.28	0.29
	Kokanee	Stream	Columbia River	36	881 (809–966)	0.29	0.30
Kootenay Lake	Kokanee	Shore (West Arm)	Columbia River	48	610 (582–642)	0.29	0.28
	Kokanee	Stream (West Arm)	Columbia River	48	838 (783–901)	0.29	0.29
	Kokanee	Stream (North Arm)	Columbia River	24	2,601 (1,843–4,414)	0.30	0.31
Tchesinkut Lake	Kokanee	Shore	Skeena River	36	2,422 (2,021–3,019)^a^	0.32	0.30
	Kokanee	Stream	Skeena River	36	–	0.31	0.30
Anderson Lake	Kokanee	Black	Fraser River	24	7,618 (3,270–infin)	0.32	0.32
Seton Lake	Kokanee	Black	Fraser River	23	1,871 (1,486–2,525)	0.32	0.31
Portage Creek	Anadromous	Stream	Fraser River	25	926 (792–1,113)	0.32	0.32

a Tchesinkut Lake samples were pooled for this analysis given the lack of structure in this location.

### RADseq Genotyping

We constructed 14 novel RADseq libraries, each consisting of between 24 and 36 pooled, individually labeled *O. nerka* individuals (*n* = 408 total) following Baird et al. (2008) as modified in Lemay and Russello (2015b). Genomic DNA was extracted using the NucleoSpin Tissue Kit (Macherey Nagel) following the manufacturer’s suggested protocol with the addition of RNase A (Qiagen). For each individual sample, 500 ng of DNA was digested using the SbfI restriction enzyme (New England BioLabs Inc.). The barcodes used were six nucleotides in length, and each differed by at least two bases (Hohenlohe et al. 2010; Miller et al*.* 2012). During the library preparation, a sonicator (Bioruptor NGS; Diagenode) was used to shear DNA strands to a mean length of ∼500 bp, and a targeted fragment-size selection device (Pippin PrepTM; Sage Science) was used instead of gel extractions to isolate DNA fragments between 350 and 600 bp in length. One full lane each of Illumina HiSeq 2000 single end 110-bp sequencing was carried out for the 14 RADseq libraries at the McGill University and Génome Québec Innovation Centre, Montréal, Canada.

Previously collected RADseq data from two identically prepared and sequenced Illumina libraries obtained from Okanagan Lake (Lemay and Russello 2015b) were also included and reanalyzed from the raw data including both shore- (*n* = 48) and stream-spawners (*n* = 48) (Lemay and Russello 2015a). All reads were trimmed, filtered, and analyzed using the STACKS pipeline (Catchen et al. 2013) in order to create catalogues of comparable SNP loci. Initially, the PROCESS_RADTAGS module was used to separate reads by their barcode, remove low-quality reads (Phred quality score < 10), trim all reads to 100 bp in length, and remove any reads that did not contain the SbfI recognition sequence. Next, the USTACKS module was used for the de novo assembly of raw reads into RAD tags. The minimum number of reads to create a stack was set at 3 (-m parameter in USTACKS), and the maximum number of pairwise differences between stacks was 2 (-M parameter in USTACKS). A catalogue of RAD tags was then generated using six *O. nerka* individuals from each putative population in CSTACKS. The distance allowed between catalogue loci (-n in CSTACKS) was increased to 1, after different trials were run to ensure loci were not inaccurately called as separate stacks. The execution of these components was accomplished using the STACKS denovo_map.pl script; in running this script, the optional -t flag was used to remove highly repetitive RAD tags during the USTACKS component of the pipeline. Following assembly and genotyping, the data were further filtered to maximize data quality. Using the POPULATIONS module, we retained only those loci that were genotyped in ≥80% of individuals and had a minor allele frequency ≥0.05 and a minimum stack depth of 10 (-m in POPULATIONS) for each individual. Genotypic data were exported from STACKS in GENEPOP format (Raymond and Rousset 1995) and converted for subsequent analyses using PGD SPIDER v. 2 (Lischer and Excoffier 2012).

Given the genome duplication event in the history of Pacific salmonid evolution, genomic samples are expected to contain a high proportion of paralogous sequence variants (PSVs); optimization of assembly parameters in salmonids is a fine balance between separating PSVs from their functional genes while not overly splitting informative variation. In general, we took an approach that was more likely to remove PSVs at the expense of potentially separating truly divergent sequence variants into separate loci. Following previous studies (Hecht et al. 2012; Gagnaire et al. 2013; Lemay and Russello 2015b; Larson et al. 2017), we removed all loci that displayed significant deviation from Hardy–Weinberg equilibrium (HWE) as assessed using the method of Guo and Thompson (1992) implemented in the R package DIVERSITY (Keenan et al. 2013). Significance levels were adjusted for multiple comparisons using a 5% false discovery rate and the method of Benjamini and Yekutieli (2001). This analysis examined HWE in each of the 14 populations; to be removed from the data, a locus had to show significant deviations from HWE in at least three populations. This filtering provided a looser stringency for merging stacks, while still controlling for PSVs. For the outlier analyses, we retained multiple SNPs in each tag as per Nichols et al. (2016) to ensure we did not lose potentially informative loci, however, for other analyses as noted below, we retained only the first SNP per tag to decrease linkage disequilibrium across samples.

RAD tags were mapped to the female sockeye salmon linkage map of Larson et al. (2016) and the RAD tags identified in sockeye salmon ecotypes in Nichols et al. (2016) and Larson et al. (2017) using BLAST-n as implemented in GENEIOUS (Kearse et al. 2012). We accepted any hit that was lower than E-25, and at least E-3 lower than any other alignment. See electronic supplementary materials for more details.

### Demography, Diversity, and Population Structure

We calculated locus- and population-specific estimates of observed (*H_o_*) and expected heterozygosity (*H_e_*) using a putatively neutral-only data set (all outliers removed, one SNP per retained RAD tag) as implemented in ARLEQUIN (Excoffier and Lischer 2010). Although no specific test was undertaken to identify purely neutral loci, eliminating all loci that exhibited significance in any of the outlier tests should remove most high leverage data points, providing a data set that reasonably estimates the overall neutral genetic differentiation between populations. Effective population sizes were estimated using the linkage disequilibrium method implemented in NEESTIMATOR (Do et al. 2014).

We investigated the number of populations (or clusters) represented in our entire sampling using FASTSTRUCTURE (Raj et al. 2014) and the putatively neutral SNP data set, default parameters, a logistic prior, and *K* from 1 to 16. The appropriate number of model components that explained structure in the data set was determined using the *chooseK.py* function (Raj et al. 2014); in this heuristic the optimal number of model components (*K*) is the minimum number of populations that have a cumulative ancestry contribution of at least 99.99%. In order to test for unrecognized substructure in the global FASTSTRUCTURE analysis, we grouped populations according to their cluster membership and repeated the above analyses on the reduced data sets as recommended by Pritchard et al*.* (2000). Results for the identified optimal values of *K* were visualized using DISTRUCT (Rosenberg 2003). We further calculated both locus-specific and population-wide *F_ST_* (Weir and Cockerham 1984) using DIVERSITY (Keenan et al. 2013), and reconstructed a 2D NeighbourNet network (Bryant and Moulton 2004) based on the pairwise putatively neutral-only population *F_ST_* values as implemented in SPLITSTREE 4.0 (Huson and Bryant 2006).

### Outlier Loci Detection and Annotation

Due to the limitations of differentiation-based methods and the potentially high false positive rates when looking for outlier loci under divergent selection (Vilas et al. 2012; De Mita et al. 2013), we utilized two distinct approaches: 1) an *F_ST_* based outlier approach between a priori ecotype pairs implemented in BAYESCAN (Foll and Gaggiotti 2008); and 2) an approach that identifies loci whose allelic divergence is significantly associated with each of the principal components within a mixed data cloud implemented in PCADAPT (Luu et al. 2017). This has the advantage of simultaneously identifying population structure and identifying the loci that are excessively linked to this structure, which may therefore by under differential selection between clusters. BAYESCAN was run first comparing ecotype paired populations (shore- and stream-spawning kokanee from within the same lake, and kokanee and anadromous sockeye salmon population pairs from the same catchment). Within Kootenay Lake this outlier detection was only carried out between shore- and stream-spawning populations in the West Arm of Kootenay Lake; North Arm individuals were excluded from this analysis, as spatial structure in this system has the potential to mask detection of candidate loci. In addition, given the low overall genomic divergence between Okanagan Lake shore- and stream-spawning kokanee, Kootenay Lake West Arm shore- and stream-spawning kokanee, and Anderson and Seton Lakes kokanee (*F_ST_ *≈ 0.01; see Results), we treated each of these as single lake populations for the purposes of *F_ST_* outlier tests between kokanee and anadromous sockeye salmon pairs. We also used BAYESCAN to evaluate combined ecotype data sets: 1) all shore-spawning kokanee versus all stream-spawning kokanee, and 2) all Okanagan catchment kokanee (Okanagan Lake, Wood Lake, Skaha Lake) versus Okanagan river anadromous sockeye salmon. For each analysis, BAYESCAN was run using 10,000 output iterations, a thinning interval of 10, 20 pilot runs of length 10,000, and a burnin period of 10,000, with prior odds of the neutral model of 10. We recorded all loci with a *q* value of 0.2 or less—which equates to a false discovery rate of 20%. *Q* values are far more stringent than *P* values in classical statistics as they are adjusted for the false discovery rate given multiple comparisons, rather than the individual false positive rates in each comparison (Storey and Tibshirani 2003).

We also conducted hierarchical outlier detection on the complete data set and on reduced subsets of the data (within each lake and/or catchment) as implemented in PCADAPT (Luu et al. 2017). The number of Principal Components retained (*K*) for each analysis was determined by the graphical approach based on the scree plot (Jackson 1993) as recommended by Luu et al. (2017). We recorded all loci with a *q* value of 0.2 or less, and identified the principal component associated with each outlier. Within each lake-level analysis, we also identified potentially misidentified individuals using the clustering output.

In addition to mapping to the female sockeye salmon linkage map (Larson et al. 2016) and the sockeye salmon RAD tags (Nichols et al. 2016; Larson et al. 2017) described earlier, all loci identified as outliers in either analysis were compared with published loci on Genbank using BLAST-n in order to identify nearby genes. We also used BLAST-n specifically in the *Salmo salar* and *O. mykiss* genomes to map each outlier locus, accepting any hit that was lower than E-25, and at least E-3 lower than any other alignment. See electronic supplementary materials for more details.

### Island of Divergence Mapping

We used a Gaussian kernel smoothing technique (Hohenlohe et al. 2010; Larson et al. 2014, 2017) to identify putative islands of divergence based on the *F_ST_* of loci that could be placed on the sockeye salmon linkage map of Larson et al. (2016). We used a window size of 5 cM and a stepwise shift of 1 cM for this analysis, and *F_ST_* values were weighted according to their window position as described by Gagnaire et al. (2013). These values were selected based on those used by Larson et al. (2017), which had been optimized for this particular linkage map, and all analysis settings matched those used by Larson et al. (2017): 1,000× sampling for each window, increasing to 10,000× for windows over the 90th percentile, and with 10,000 bootstrap replicates. Along with the formal tests for islands of divergence, we compared the locations of outlier loci identified in different pairwise tests to see if there were patterns of shared genomic regional divergence beyond the sharing of the same SNP.

### Linkage Disequilibrium

We calculated linkage disequilibrium (LD) between all markers identified as potentially under divergent natural selection by either outlier tests across all comparisons, using the R package LDheatmap (Shin et al. 2006).

## Results

### RADseq Genotypic Data and Alignment

A total of 2,269,727,378 Illumina single-end reads were processed from the 16 libraries, with 73% (1,655,919,400) of these reads retained after quality filtering and barcode assignment to individual. Each individual had on average 2.88×10^6^ reads (1.96E6–3.96E6 95% CI), with a median of 2.98×10^6^ reads retained after quality filtering. Following RAD sequencing, processing, and filtering, we collected genotypic data at 8,436 SNPs (30× average coverage) for 504 individuals representing anadromous sockeye salmon and resident kokanee ecotypes from seven lakes and two rivers across three historically defined catchments in BC (fig. 1, table 1 and supplementary data file S1, Supplementary Material online). After filtering for within population deviations from Hardy–Weinberg equilibrium, this number was reduced to 7,347 SNPs across 6,568 RAD tags (supplementary data file S1, Supplementary Material online). Of these tags, 41 (0.62%) contained three SNPs per tag, 738 (11.2%) contained two SNPs per tag, and 5871 (89.4%) contained only one SNP. We removed 32 individuals due to low coverage (<50% SNPs retained), giving a final data set of 472 individuals across the 14 sampling units (table 1).

Of the 6,568 retained RAD tags in our study, 62% (*n* = 4,074) were unambiguously mapped to the data sets of Larson et al. (2016, 2017), with 71% (*n* = 2,888; 44% of our total RAD tags) of these situated on their sockeye salmon linkage map (supplementary data file S2, Supplementary Material online). Furthermore, 1,714 (26%) of our tags matched those retained by Nichols et al. (2016), equating to 66% of the 2,593 RAD tags used in their study (supplementary data file S1, Supplementary Material online).

### Demography, Diversity, and Population Structure

Estimates of population-level observed and expected heterozygosity based on 6,234 putatively neutral loci [outliers removed (see below); one SNP per RAD tag] were virtually identical across all ecotypes and locations (0.29–0.32; table 1). Effective population sizes varied by an order of magnitude between populations (242–9,851; table 1), with no clear trends observed by spawning location or ecotype (table 1).

**Fig. 2.**
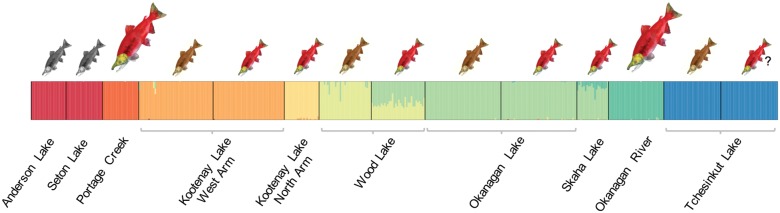
—FASTSTRUCTURE (Raj et al. 2014) plot based on genotypic data at 6,234 putatively neutral SNPs showing the proportion of cluster membership at *K *=* *8. Vertical bars indicate individuals, colors represent proportion of cluster membership. Size and color of *Oncorhynchus nerka* images as in figure 1.

The FASTSTRUCTURE analysis based on the putatively neutral data set provided evidence for eight distinct clusters in our data, grouping together Anderson and Seton Lake black kokanee, Kootenay West Arm shore- and stream-spawning kokanee, Okanagan Lake shore- and stream-spawning kokanee combined with Skaha Lake kokanee, and Tchesinkut Lake shore- and stream-spawning kokanee (fig. 2). In this global analysis, Wood Lake included a large contribution from Okanagan/Skaha Lake, with Skaha Lake also showing admixture with Okanagan River sockeye salmon. The presence of substructure in the data was further explored in each of these systems individually; only Wood Lake revealed two distinct clusters corresponding to shore- and stream-spawning kokanee when analyzed independently (supplementary fig. S1, Supplementary Material online).

**Fig. 3.**
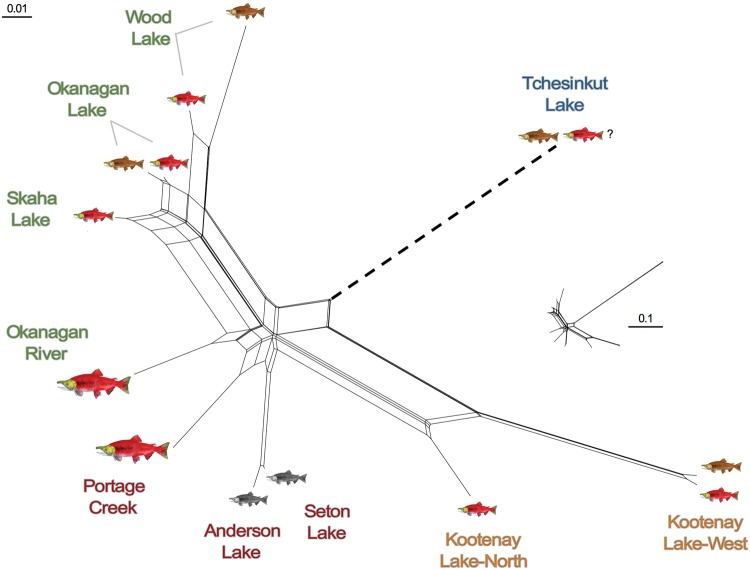
—Two-dimensional NeighbourNet (Bryant and Moulton 2004) population network based on FST (Weir and Cockerham 1984) calculated using genotypic data at 6,234 neutral SNPs. The length of the Tchesinkut lake branch is shortened to enable a better comparison of the other branches; the relative length of the whole network shown inset. Size and color of *Oncorhynchus nerka* images as in figure 1. Color of the text for each place name denotes drainage (green = Columbia River, red = Fraser River, blue = Skeena River). Kootenay Lake kokanee may potentially be historically linked to either the Columbia or Fraser drainages, and indicated in orange.

The phylogenetic network based on the putatively neutral data set revealed a clear separation between the historical Skeena River drainage site (Tchesinkut Lake) and all the Columbia-Fraser drainage lakes and rivers (*F_ST_*=0.37–0.45) (fig. 3 and supplementary table S1, Supplementary Material online). Kootenay Lake kokanee exhibited significant population structure between the North Arm and West Arm populations (*F_ST_*=0.13), which were both more closely related to Fraser River drainage sites (Anderson Lake/Seton Lake kokanee and Portage Creek anadromous sockeye salmon) than to the Columbia River drainage sites sampled in the Okanagan Basin (Okanagan, Wood, and Skaha Lakes). At a finer level, shore- and stream-spawning populations sampled in the same lake (Okanagan, Wood, Kootenay, Tchesinkut) were more closely related to each other than to corresponding ecotypes in other lakes without exception (fig. 3). These paired kokanee ecotype samplings exhibited widely differing levels of intralake divergence and in some cases, possible introgression. Wood Lake shore- and stream-spawning kokanee were moderately divergent (*F_ST_*=0.060), whereas Okanagan Lake shore- and stream-spawning kokanee and Kootenay Lake West Arm shore- and stream-spawning kokanee were considerably less so (*F_ST_*=0.008; supplementary table S1, Supplementary Material online and fig. 3). There was minimal differentiation between kokanee sampled on the shore and at the mouth of the stream in Tchesinkut Lake (*F_ST_*=0.003; supplementary table S1, Supplementary Material online and fig. 3).

The PCADAPT analyses both highlight population structure and simultaneously identify outlier loci most highly associated with this structure. Here, we will present the population structure indicated by these analyses (see below for outlier analysis). We found the optimal number of principal components to be nine for the full data set. For most of the principal components retained, geographic structure was revealed rather than ecotype differentiation (supplementary fig. S2, Supplementary Material online). The first two principal-components match the structure indicated by the phylogenetic network—PC1 (15.07% of variation) separating Tchesinkut Lake kokanee from all other populations, PC2 (10.6% of variation) separating Kootenay Lake kokanee with all other populations (supplementary fig. S2*A*, Supplementary Material online). PC3 (3.58% of variation) separates the current Fraser River populations (Anderson and Seton Lake Kokanee, Portage Creek anadromous sockeye salmon) from the other populations (supplementary fig. S2*B*, Supplementary Material online). PC4 (2.01% of variation) is more complex, with populations spread out, though with a less clear pattern (supplementary fig. S2*B*, Supplementary Material online). There is some separation of shore/stream-spawning populations on this axis including: Wood Lake shore- and stream-spawning kokanee, Anderson/Seton Lake shore-spawning kokanee, and Okanagan River stream-spawning anadromous sockeye and Skaha Lake stream-spawning kokanee; however, other shore/stream-spawning population pairs are not separated. PC6, PC7, PC8, and PC9 each separated one population from all of the others (Kootenay North Arm kokanee, Portage Creek anadromous sockeye salmon, Wood Lake stream-spawning kokanee, and Skaha kokanee, respectively; supplementary fig. S2*C*–*E*, Supplementary Material online).

The PCADAPT results were more interpretable relating to ecotype divergence when conducting analyses at the lake level. Within the Anderson/Seton Lake and Portage Creek system, two principal components were optimal, with PC1 (6.78% of variation) separating Portage Creek anadromous sockeye salmon from the two kokanee populations, and PC2 (1.64% of variation) separating Anderson and Seton Lake shore-spawning kokanee from each other (supplementary fig. S3*A*, Supplementary Material online). Similarly, within Kootenay Lake, two principal components were optimal, with PC1 (6.3% of variation) separating the North Arm stream-spawning kokanee, from the two West Arm populations, and PC2 (1.57% of variation) separating the West Arm stream-spawning kokanee from the West Arm shore-spawning kokanee (supplementary fig. S3*B*, Supplementary Material online). Three potentially misidentified individuals were detected between the two West Arm kokanee populations. Within the Wood Lake system, two principal components were also optimal, with PC1 (5.76% of variation) separating the shore- and stream-spawning kokanee populations, and PC2 (1.68% of variation) separating off two shore-spawning individuals (supplementary fig. S3*C*, Supplementary Material online). Again, some potentially misidentified individuals were detected—two shore-spawners in the stream-spawning cluster. In all of these lake systems where samples were apparently misidentified, it is possible that these individuals are strays, or could represent sampling error, where sampled fish were either not spawning or had drifted from their spawning location. For the three remaining within-lake comparisons, it was unclear if one or two principal components were optimal, so while we display two principal components for each of these analyses, we acknowledge that potentially only the first is supported. From the Okanagan Lake analysis, PC1 (1.52% of variation) separates perfectly the shore- and stream-spawning kokanee (supplementary fig. S3*D*, Supplementary Material online). We further annotated the PC plot with the various spawning locations sampled in case there was a spatial pattern to PC2 (1.12% of variation), however, all streams and shore-spawning locations were mixed evenly along this axis, showing no detectable within-lake spatial structure beyond ecotype differentiation. From the Tchesinkut Lake analysis, no ecotype separation was evident in any principal component, with complete overlap between “ecotypes” (supplementary fig. S3*E*, Supplementary Material online). Within the Skaha Lake kokanee/Okanagan River anadromous sockeye analysis, PC1 (8.21% of variation) fully separated each ecotype (supplementary fig. S3*F*, Supplementary Material online).

### Outlier SNP Analyses

The two outlier analyses methods used evaluated different kinds of divergence—pairwise divergence between a priori groups in BAYESCAN, and a posteriori evaluations of divergence between computed clusters in PCADAPT. These analyses are therefore sometimes comparable when these two groupings matched, but incomparable (though complementary) when the principal components did not match the predefined populations. These complementary analyses therefore add different views of outlier loci and their underlying drivers.

Because the principal components identified by PCADAPT in the complete data set analyses either primarily reflected geographic differentiation (PCs 1–3) or single-population-specific outliers (PCs 6–9), we report these outlier SNPs (supplementary data file S3, Supplementary Material online), but do not analyze them in detail here. Rather, we focus on the genomic changes underlying ecotype divergence that were identified by both outlier detection approaches (BAYESCAN and PCADAPT).

**Fig. 4.**
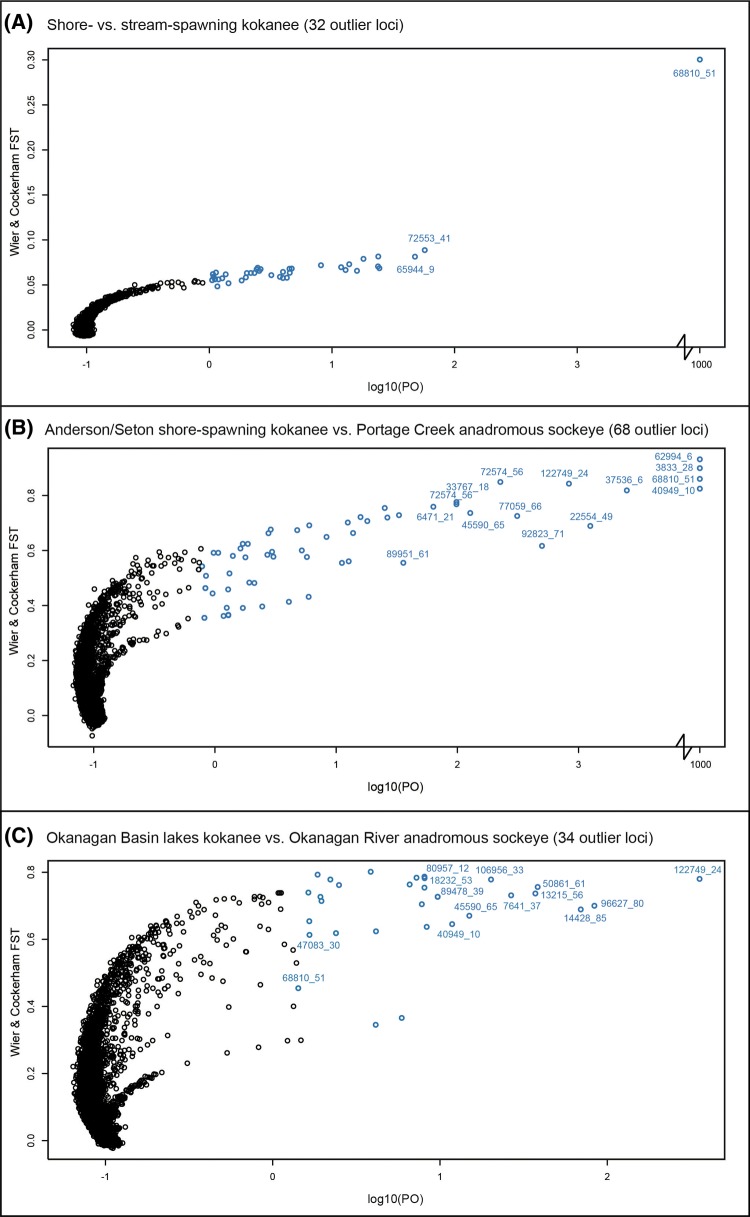
—*F_ST_* outlier plots for the three primary combined ecotype comparisons performed in BAYESCAN (Foll and Gaggiotti 2008) including: (*A*) shore- versus stream-spawning kokanee; (*B*) Anderson/Seton Lake shore-spawning kokanee versus Portage Creek anadromous sockeye salmon; and (*C*) Okanagan Basin lakes kokanee versus Okanagan River anadromous sockeye salmon. Note the disjoint *x*-axis, with loci that had a *q* value of 0.0000, and a log10(PO) of 1,000 brought in to aid visibility.

Numerous outlier loci were detected across the three primary pairwise ecotype comparisons including Anderson/Seton Lake shore-spawning kokanee versus Portage Creek anadromous sockeye salmon (*n* = 68), Okanagan Basin kokanee (Okanagan Lake, Wood Lake, Skaha Lake) versus Okanagan River anadromous sockeye salmon (*n* = 34), and all shore-spawning kokanee versus all stream-spawning kokanee (*n* = 32; fig. 4). Of these, three outliers (R40949, R68810, R122729) were shared in multiple comparisons. In particular, R68810 was identified in all three comparisons, and was notably the most significantly divergent in the shore-spawning versus stream-spawning kokanee comparison by several orders of magnitude (fig. 4).

Across these three combined comparisons, along with the four-paired shore/stream spawning kokanee comparison within lakes, and the six-paired kokanee/anadromous sockeye salmon comparisons, a total of 334 outlier loci were detected in at least one comparison across the two methods. Of these, 68 were detected in at least two different comparisons, with 12 recorded in three comparisons, 9 recorded in four comparisons, 7 recorded in five comparisons, and 3 recorded in six comparisons. Furthermore, 11 of our outlier loci were detected as outliers between sockeye salmon ecotypes in Nichols et al. (2016), and 10 were recorded as outliers between reproductive ecotypes of anadromous sockeye salmon in Larson et al. (2017) (table 2 and supplementary table S2, Supplementary Material online). Comparing all outliers detected in BAYESCAN with those in directly comparable PCADAPT analyses, 37% were identified by both analyses, 37% were identified by BAYESCAN only, and 26% were identified by PCADAPT only (supplementary data files S3–S6, Supplementary Material online).

**Table 2:** Outlier Loci Independently Detected in Three Studies of Anadromous Sockeye Salmon and/or Kokanee Across Multiple Sites and Catchments

Site	Multiple Sites	Redfish Lake	Alturas Lake	Bristol Bay
Catchment	Columbia, Fraser, Skeena Rivers (British Columbia, Canada)	Snake River (Idaho, USA)	Snake River (Idaho, USA)	Wood River (Alaska, USA)
Reference	This Study	Nichols et al. (2016)	Nichols et al. (2016)	Larson et al. (2017)
RAD tag	24343_34	64477	64477	41305
	27613_58	67582	–	–
	42694_49	–	–	86418
	54987_82	10067	–	–
	58500_65	–	–	11863
	59110_15	37622	–	–
	62994_06	84113	–	–
	68420_46	–	–	82702
	68810_51	57884	–	27165
	84183_30	–	30017	–
	86537_58	–	22082	–
	89327_44	–	–	88727
	90758_52		–	25509
	93027_29	–	–	12430
	99307_74	–	–	26600
	105177_36	–	13591	–
	114019_82	24175	24175	–
	119684_49	20138	–	–

Note.—Color shading indicates the comparison within which an outlier was detected including: shore/stream-spawning (light gray), kokanee/anadromous sockeye salmon (dark gray), and mixed shore/stream-spawning and kokanee/anadromous sockeye salmon comparisons (gray grid). Study-specific RAD tag codes are also indicated.

Shared outlier loci between pairwise comparisons were most common between Okanagan River anadromous sockeye salmon in comparisons with nearby lakes within the Okanagan Basin (Okanagan Lake, Wood Lake, and Skaha Lake) as well as between the two populations in Kootenay Lake. Seven of the outlier loci detected between Anderson and Seton Lake kokanee and Portage Creek anadromous sockeye salmon were also detected in other population comparisons (supplementary data files S4 and S5, Supplementary Material online), six of which have high confidence annotations (table 3).

**Table 3:** Annotations for High-Confidence Outlier Loci Independently Detected across the Columbia and Fraser River Catchments

RAD Tag	SNP	Accession No.	Gene	*E* Value
7641	37	NC_027312.1	RACGAP1 Rac GTPase activating protein 1	5.00E-37
18232	53	NC_027315.1	LOC106573447 hepatocyte growth factor-like	1.00E-38
40949	10	NC_027305.1	LOC106608136 heat shock protein HSP 90-alpha-like	4.00E-32
47083	30	NC_027304.1	LOC106605382 dendritic cell-specific transmembrane protein-like	1.00E-39
68810	51	NC_027308.1	LOC106610979 leucine-rich repeat-containing protein 9-like	2.00E-42
122749	24	NC_027302.1	LOC106598978 angiopoietin-related protein 4-like	1.00E-38

The common shore/stream outlier locus R68810 was independently detected in three paired kokanee ecotype comparisons (supplementary data files S4 and S5, Supplementary Material online), as well as in both Nichols et al. (2016) and in Larson et al. (2017) (table 2). This shared outlier locus had the highest differentiation in both Anderson-Seton Lakes kokanee/Portage Creek anadromous sockeye salmon (*F_ST_* = 0.83), and in Okanagan Lake kokanee shore- and stream-spawning comparisons (*F_ST_* = 0.92), and was significantly differentiated between Wood Lake kokanee shore- and stream-spawning populations (*F_ST_* = 0.43). R68810 was also detected as a highly significant outlier between two other shore-spawning kokanee and stream-spawning anadromous sockeye salmon pairs [Okanagan Lake shore-spawning kokanee/Okanagan River sockeye (*F_ST_* = 0.97), and Wood Lake shore-spawning kokanee/Okanagan River anadromous sockeye salmon (*F_ST_* = 0.90); supplementary table S2, Supplementary Material online].

### Islands of Divergence

In all three of the primary comparisons, we found regions of the genome with elevated divergence (supplementary fig. S4 and data file S7, Supplementary Material online). Due to the relatively low coverage of SNPs across this linkage map (44% of SNPs retained in our study mapped), there may be other regions with shared elevated divergence that could not reach statistical significance given the sampling level. Although statistical islands of divergence were identified, they did not generally constitute clear clusters of highly differentiated outlier loci. Only 18 of 47 outlier loci were located in the identified islands of divergence, with a maximum of only two outlier loci clustered in the same island. With the comparatively small number of loci mapped, it is perhaps unsurprising that a more densely populated map is required to reveal clear islands of divergence. Nevertheless, there were some shared islands of divergence with those reported elsewhere that may warrant further investigation.

Of particular note are putative islands of divergence shared between our comparisons and/or with other studies. Islands LG12_3 and LG12_4 were almost identical in location, with LG12_3 identified between the Anderson and Seton Lake shore-spawning kokanee and the Portage Creek anadromous sockeye salmon, whereas LG12_4 was identified in the combined paired kokanee shore/stream comparison. Similarly, LG24_1, LG24_2, and LG24_3 have overlapping regions and were identified in both the Anderson and Seton Lake shore-spawning kokanee/Portage Creek anadromous sockeye salmon comparison, and the Okanagan Basin Lakes kokanee/Okanagan River anadromous sockeye salmon comparison.

Comparing our results with those of Larson et al. (2017), we also found two shared islands of divergence. Our LG12_1 and LG12_2 overlap Larson_LG12_1 (Larson et al. 2017), one of the most prominent island of divergence between their beach-spawning and river-spawning anadromous sockeye salmon (and containing our shared R68810 outlier). Finally our LG15_1 matches Larson_LG15_1 (Larson et al. 2017), a region containing the known MHC genes. Beyond the formal identification of genomic islands of divergence, we also found several areas of the genome containing multiple outlier SNPs, but from different pairwise comparisons (supplementary table S3 and data file S2, Supplementary Material online).

### Alignment to the *S. salar* Genome and Annotation of Outlier Loci

One hundred and sixty-two outlier loci mapped to the sockeye linkage map of Larson et al. (2016) (supplementary data file S2, Supplementary Material online). Due to the substantial divergence between *S. salar* to *O. nerka* (15–20 Ma; Stearley 1992) and the repetitive nature of their genomes, only BLAST-n hits lower than E-25, and at least E-3 lower than any other hit were accepted to ensure a conservative and accurate annotation. One hundred and fifty outlier loci unambiguously aligned within the Atlantic salmon (*Salmo salar*) genome, including the common shore-stream outlier R68810 (supplementary data file S5, Supplementary Material online). In addition to the specific annotations of these individual loci detailed in supplementary data file S5, we detected three instances where two RAD tags overlapped by 4 bp (R50861/R89478, R95009/R97203, R88765/R17286) flanking the same restriction site. These RAD tag pairs (and the associated outlier SNPs) therefore should each be treated as a single locus. More distant potential linkage was also detected based on the alignment with the *S. salar* genome, with R40949 and R74283 ∼367 kb away from each other in the *S. salar* genome. These two loci were among the most commonly identified anadromous sockeye/kokanee outlier loci in our comparisons (four comparisons and five comparisons, respectively), suggesting they are potentially linked to the same genomic region under differential selection, and within the same island of divergence—though precisely where on the sockeye salmon linkage groups remains uncertain as neither mapped to the linkage groups of Larson et al. (2016).

Our calculation of linkage disequilibrium identified the above-mentioned pairs of closely linked loci, as well as a small number of apparently linked loci pairs (supplementary fig. S5, Supplementary Material online). Because many of these loci were fixed in some populations, LD calculations could not be carried out in all cases, however, we present all of the within population calculations for comparison (supplementary fig. S5, Supplementary Material online).

## Discussion

Our results offer important insights into the evolutionary history of *O. nerka*, inferring genomic mechanisms underlying adaptive divergence and identifying specific genes that exhibit parallel patterns across multiple ecotype-pairs sampled from different catchments. Furthermore, the connectibility of our genotypic data with those from previously published studies employing SbfI RADseq enable us to explore the generality of these mechanisms and loci at a broader scale.

### Evolutionary History of Sockeye Salmon

Although our study was not designed to provide a comprehensive investigation of *O. nerka* evolutionary history in BC, the patterns of divergence we observed between sampled populations based on 6,234 putatively neutral SNPs reinforce findings from previous studies (Taylor et al. 1996). We found a clear separation between the historical Skeena River drainage population sampled in Tchesinkut Lake and all kokanee and anadromous sockeye salmon populations sampled from the Columbia-Fraser River drainages, consistent with the multiple glacial refugia hypothesis set forth by Taylor et al. (1996). Within the Fraser River drainage, Anderson and Seton Lake black kokanee were most closely related to Portage Creek anadromous sockeye salmon, as previously found (Moreira and Taylor 2015), and kokanee from these two lakes were differentiable from each other based on outlier loci. Within the Columbia River drainage, the close relationships between kokanee (Okanagan, Wood, and Skaha Lakes) and anadromous sockeye salmon in the Okanagan River basin indicate these populations evolved from a common source and/or that there has been ongoing gene flow. The close connection between Skaha Lake kokanee, Okanagan Lake kokanee, and Okanagan River anadromous sockeye salmon exemplifies these patterns. Skaha Lake kokanee spawn in the channel between Okanagan Lake and Skaha Lake, and Okanagan River anadromous sockeye salmon historically spawned in the same channel, before becoming isolated following construction of McIntyre Dam in 1916 (reviewed in Veale and Russello 2016). In contrast, Kootenay Lake kokanee populations were highly divergent from the nearest populations sampled in the Columbia River drainage, more similar to the Anderson and Seton Lake kokanee. Taylor et al. (1996) similarly found kokanee in Kootenay Lake and nearby lakes to have a closer affinity to the Fraser River drainage rather than populations sampled in the Columbia River drainage, possibly due to changes in river connection; our results based on the large SNP data set are consistent with this hypothesis.

At a finer level, shore- and stream-spawning kokanee ecotypes in each lake are sister taxa, highlighting the recurrent divergence of reproductive ecotypes. In all but one lake, kokanee ecotypes were genetically distinct from each other. The only shore- and stream-spawning *O. nerka* that could not be genetically differentiated by neutral and/or outlier loci were in Tchesinkut Lake, consistent with the findings of Frazer and Russello (2013). The vast majority of individuals spawn on the shores of an island in Tchesinkut Lake, where the “shore-spawners” were originally sampled in 2010 (Frazer and Russello 2013). The “stream-spawners” were sampled at an outflow and Drew Creek, however, the latter spawning activity only occurred in this small creek once government personnel cleared the mouth allowing kokanee access (Frazer and Russello 2013). These results further suggest there may not be distinct ecotype populations in this lake, though additional molecular and behavioral studies will be necessary to investigate this to rule out recent divergence or on-going gene flow.

### Genomic Bases for Ecotype Divergence

Identifying the drivers underlying parallel ecological divergence is an important step toward understanding the balance between deterministic and stochastic processes of evolution, and speciation. There have been a large number of recent studies into the genomic basis for parallel ecotype evolution across a range of taxa (Roesti et al. 2014; Milano et al. 2016; Ravinet et al. 2016; Westram et al. 2016; Kusakabe et al. 2017; Rougemont et al. 2017). In most of these studies, the majority of outlier loci were not shared in comparisons between independent ecotype pairs, with demographic history and ongoing connectivity explaining a majority of shared divergence (Westram et al. 2016; Rougemont et al. 2017).

Similarly, the majority of divergence between populations in our study was between different catchments and regions, rather than between ecotypes. Although some of this differentiation may be the result of divergent selection, it is likely that genetic drift between these isolated populations is primarily responsible for this pattern, especially given the lower estimated effective population sizes in some locations (table 1). We did, however, identify some highly differentiated genomic regions and outlier SNPs that may be under divergent selection between ecotype pairs, with some islands of divergence and outlier loci observed in multiple pairwise ecotype comparisons. We identified common outlier loci (e.g., R68810, R40949, R122749) with patterns of differentiation that were shared between ecotype pairs, both in terms of extent and direction, and present in multiple catchments. This pattern is suggestive of genetic variation that was present in ancestral populations that has been independently and divergently selected in multiple instances under similar selective regimes, or that have spread between adapted populations through introgression (Le Moan et al. 2016), potentially via other populations (e.g., transporter hypothesis; Welch and Jiggins 2014).

Along with these clear shared outlier loci between catchments, we found genomic regions in many kokanee-anadromous sockeye salmon pairs where an outlier SNP or island of divergence were highlighted, but in each comparison, the exact SNPs showing differentiation were different. There are multiple hypotheses that may explain this pattern. Potentially, differences in heterozygosity in loci in these regions can lead to differently detected outlier loci. This pattern may also indicate that a gene(s) in this region may be repeatedly differentially selected between ecotypes through novel beneficial mutations occurring in each instance, or that novel neutral SNPs have arisen in each population linked to pre-existing variants that have since become targets for selection. As with studies of this nature, further validation is required in order to provide more direct evidence that outlier behavior reflects signatures of selection as well as to make any firm conclusions on the nature of that selection. These validation steps would include: 1) assessing these SNP markers in an independent set of individuals from these same lakes to confirm that they are indeed real outliers in these systems; 2) evaluating their level of differentiation in other systems; and 3) by sequencing or genotyping larger flanking regions to investigate the size and potential functional changes in nearby genes or regulatory regions. The first of these steps provides evidence that the outlier SNP is indeed linked to ecotype divergence within the system (Westram et al. 2016), whereas the latter two steps can begin to investigate the potential causal mechanisms and evolutionary history. Finally, once a probable causative gene associated with divergent phenotypes is identified, a final validation step could explore gene knockouts or knockdowns or CRISPR-Cas editing (Edvardsen et al. 2014; Liu and Lin 2017). We have already begun validating some of these outliers on independent samples: comparing Skaha Lake kokanee with Okanagan River anadromous sockeye (R122749, R40949, R7641, R47083, R18232; Veale and Russello 2016), and for the shore/stream spawning locus R68810 in both independent samples from the same lakes, and in numerous other catchments (Veale and Russello 2017).

### Islands of Divergence

Outlier SNPs detected within one pairwise comparison and located adjacent to each other on the sockeye salmon linkage map are potentially closely physically linked, and thus may constitute islands of divergence exhibiting the same signal of differential selection.

Our formal tests for islands of divergence identified a number of potential genomic regions with increased divergence. Although we used identical methods and linkage maps to Larson et al. (2017), the number of loci retained for our analyses was significantly fewer, as not all of our loci were able to be mapped unambiguously to their linkage map. The number of loci genotyped may affect the ability to identify islands of different sizes, with denser sequencing tending to reveal more and smaller islands (Nosil et al. 2012; Soria-Carrasco et al. 2014). In our study, the number of SNPs per island was on average slightly lower, and the percentage of outliers in the islands of divergence was likewise lower than those recorded by Larson et al. (2017). This may be a consequence of the lower SNP coverage, differing scopes of the studies, and/or varying levels of population differentiation present in our respective systems.

Both the similarities and differences in location of islands of divergence between our results and those of Larson et al. (2017) are of interest and may show various patterns of divergent evolution or rapid genetic drift. We detected no evidence of divergence at the large island on linkage group 13 near the TULP4 gene, indicating that perhaps this is a novel mutation in the anadromous sockeye salmon in Alaska, rather than a more universal region underlying ecotype differentiation. We did, however, find some overlap in islands of divergence on linkage groups 12 and 15, which may represent genomic regions that are rapidly evolving in each population, are experiencing higher purifying selection, have decreased recombination, or are more generally under divergent selection among ecotypes. Around the R68810 locus there was an island of divergence identified both in our comparisons, and in Larson et al. (2017). Although there are many potential mechanisms that can create islands of divergence, when these are driven by adaptive divergence, strong selection and high gene flow are hypothesized to create fewer islands that vary in size, but display high differentiation. In contrast, weaker selection and low gene flow are hypothesized to create numerous small islands displaying lower differentiation (Via and West 2008; Nosil et al. 2009). In the study of beach- versus stream- versus river-spawning anadromous sockeye salmon in Alaska (Larson et al. 2017), few small islands of divergence with generally low divergences were found, suggesting high selective pressure acting on small segments of the genome under high gene flow. Our results showed similar numbers and sizes of islands of divergence, but with higher divergence outside these areas. Although important differences in both the geographic scope and density of SNPs between our study and that of Larson et al. (2017) may help account for these patterns, it seems likely that there is far greater isolation between ecotypes in our BC systems, leading to the effects of genetic drift being more prominent across the genome.

While we have primarily discussed adaptive mechanisms that may lead to both islands of divergence and shared outlier loci, background purifying selection linked to these loci or regions of the genome with low recombination may also create these patterns (Burri 2017). We present these regions and SNPs as areas of interest for future investigation, as further sequencing, breeding, and QTL studies may be able to tease apart potential underlying mechanisms and also help identify any divergently selected genes in these regions if they do exist.

### Anadromous Sockeye/Kokanee Outlier Loci

Although the “kokanee phenotype” can be defined as a migratory ecotype, it constitutes a range of morphological, physiological, and behavioral differences compared with anadromous sockeye salmon; therefore understanding the effects of genes linked to this phenotype is challenging. In similar studies of ecotypes in salmonids (Larson et al. 2016; McKinney et al. 2016; Leitwein et al. 2017), stickleback (Roesti et al. 2015), and sculpins (Dennenmoser et al. 2017), patterns of differentiation are often observed scattered across the genome, with multiple genes implicated in affecting polygenic traits. The outlier loci described in this study provide potential clues as to some of the genes that may underlie these traits, though further research is required to ascertain the specific changes linked to each SNP, and how these genes affect phenotype. We used a relatively high false discovery rate (FDR) of 20% for each of our comparisons as our sample sizes per population were moderate, and we wanted to: 1) include all potential outlier loci as hypotheses for loci that are highly differentiated for comparison between independent tests; and 2) enhance confidence that our neutral data reflect genome-wide patterns. Within this framework, the identification of outlier loci recorded across multiple independent comparisons provides partial validation and highlights differentiated loci worthy of further investigation. Many of these commonly shared outlier SNPs lie within, or adjacent to, genes with functions that could be related to adaptive traits.

Here, we briefly discuss outlier loci that exhibited the greatest differentiation for which we have reliable annotations and that were partially validated through their shared detection across historically isolated drainages (table 3). There were, however, hundreds of outlier SNPs detected that may be of interest for further investigation. It is also noteworthy that kokanee and anadromous sockeye salmon comparisons in the Fraser River drainage were restricted to the unique black kokanee ecotype, whereas the Columbia River drainage comparisons involved more typical stream- and shore-spawning kokanee, rendering our estimates of shared outliers associated with migratory ecotypes between systems to be highly conservative. Although we describe the annotated or nearest gene(s) for each of these SNPs, it remains possible that other nearby genes or regulatory sequences are the actual targets of selection.

R122749 contained the most commonly shared kokanee/anadromous sockeye salmon outlier SNP, detected in five population comparisons and in both major river catchments. In both anadromous sockeye salmon populations, the C allele at this SNP was found at >90% frequency in each population, whereas for all kokanee populations the C allele was found at <10% frequency. R122749 maps to the angiopoetin-4 gene that regulates angiogenesis and the development of perivascular cells, along with regulating adipocyte differentiation and metabolism (Zhu et al. 2012). This gene is differentially selected and up-regulated in Tibetan chickens as a probable adaptation to hypoxia (Zhang et al. 2016), and dissolved O_2_ could constitute a target for differential selective pressure between kokanee and anadromous sockeye salmon given the variation in the environments they inhabit throughout their life cycle.

R40949 contained an outlier SNP in four kokanee/anadromous sockeye salmon comparisons, and is located in HSP90, a heat shock gene that regulates a variety of cellular processes in response to environmental temperature (Pratt and Toft 2003). In anadromous sockeye salmon populations, the G allele at this SNP was found at ∼95% frequency, whereas kokanee populations averaged more uniform frequencies (∼47% A allele, ∼53% G allele). Sockeye salmon populations display differences in thermal tolerance over both broad and fine spatial scales, and thermal stress has been identified as a major selective pressure (Eliason et al. 2011). Since kokanee and anadromous sockeye salmon experience greatly differing thermal regimes throughout their lifecycle, this gene constitutes a strong candidate to be differentially selected. QTL studies in Arctic char (*Salvelinus alpinus*) have previously demonstrated HSP90 divergence between thermal-tolerant and thermal-sensitive individuals (Quinn et al. 2011). Similarly, divergent selection at HSP90 has been demonstrated in sympatric *Anolis* lizards that occupy different thermal environments (Akashi et al. 2016). Interestingly, ubiquitin, which is often involved in the same pathways as and interacts with HSP90, has also been linked to thermal tolerance in Arctic char (Quinn et al. 2011), and ubiquitin conjugating enzyme was one of the other strong outliers recorded between Anderson/Seton Lake kokanee and Portage Creek anadromous sockeye salmon (R111962).

R7641 contained an outlier SNP in four kokanee/anadromous sockeye salmon comparisons and annotates to the Rac GTPase Activating Protein 1 (RACGAP1) gene. In anadromous sockeye salmon populations, the T allele at this SNP was found at a frequency of ∼71%, whereas in kokanee population, the allele frequencies flipped (∼26% T allele). RACGAP1 has been shown to regulate sperm maturation in rainbow trout (Sambroni et al. 2013) and to be involved in complex signaling pathways that respond to varying water quality (Gagné and Cyr 2014).

R47083 is located in the dendritic cell-specific transmembrane protein (DC-STAMP) and was significant in three kokanee/anadromous sockeye salmon comparisons. In anadromous sockeye salmon populations, the T allele at this SNP was found at >71% frequency, whereas kokanee populations possessed more uniform frequencies (∼54% C allele, ∼46% T allele). This protein is related to bone redevelopment and haematopoesis regulation, along with having functions in immune homeostasis (Kobayashi et al. 2016). Variants in the DC-STAMP gene have been linked to size variation in humans, due to its regulation of bone remodeling activity (van der Valk et al. 2015). Given the different morphology and size of kokanee compared with anadromous sockeye salmon, this may be a candidate for divergent selection.

R18232 contained an outlier SNP that was significant in three kokanee/anadromous sockeye salmon comparisons and maps to the hepatocyte growth factor (HGF) gene, which regulates embryogenesis, organogenesis, and tissue regeneration by regulating cell growth and morphogenesis, and plays a central role in angiogenesis (Fajardo-Puerta et al. 2016). Anadromous sockeye salmon populations exhibited roughly equal frequencies of the G (∼45%) and T (∼55%) alleles at this SNP, whereas kokanee populations largely possessed the G allele (∼90%). Previous QTL studies have highlighted this gene as being associated with altering growth rates in turbot (*Scophthalmus maximus*) (Robledo et al. 2016).

There are many other SNPs identified in this study that are worthy of further investigation. For instance, a total of 24 RAD tags (R7641, R11041, R13215, R14428, R18232, R22663, R24343, R40494, R45590, R46876, R47083, R50861, R59110, R61789, R68424, R68810, R74283, R80957, R89478, R93027, R96627, R106956, R114019, R122749) contained outliers independently in at least three population comparisons. Of these, R59110 was also recorded as an outlier between *O. nerka* ecotypes in Nichols et al. (2016), and maps to growth differentiation factor 3 (GDF3), another gene with strong potential for divergent selection between kokanee and anadromous sockeye salmon given its important role in ocular and skeletal development (Ye et al. 2010).

We also note that outlier loci not shared between comparisons may likewise be of interest, as they may be linked to novel mutations responsible for the complex and divergent kokanee and anadromous sockeye salmon phenotypes. For instance, R3833, R62994, R22554, and R37537 are all extremely highly differentiated between Anderson Lake and Seton Lake black kokanee and Portage Creek anadromous sockeye salmon. The unusual spawning behaviour and selective environment associated with the “black kokanee” phenotype may potentially be linked with changes in the regions near these loci. Of particular note, R62994 was also the most significant outlier in Nichols et al. (2016). All outlier loci are given in supplementary data file S5, Supplementary Material online, along with their alignments to the *Salmo salar* genome where possible.

### *The “Shore-Stream” Locus* (R68810)

The extremely high differentiation of the outlier SNP in R68810 between shore-spawning kokanee and stream-spawning kokanee and anadromous sockeye salmon in both the Columbia River and Fraser River drainages highlights the probable importance of a gene in this region influencing ecotype divergence. This locus was also identified as a high-confidence outlier by Larson et al. (2017) in comparisons between lake- and river-spawning anadromous sockeye in Alaska, and by Nichols et al. (2016) in a comparison between shore-spawning anadromous sockeye salmon and stream-spawning kokanee in the Snake River catchment, further validating its potential significance. In our study, the only shore-/stream-spawning ecotype pair that did not show any divergence at this SNP was in Tchesinkut Lake, and as discussed earlier, there is no genetic evidence associated with ecotype differentiation in this location. In the West Arm of Kootenay Lake, while patterns of variation at this SNP were recorded in the same direction as observed in other comparisons (i.e., the G allele with higher prevalence in the shore-spawning population), differentiation was less significant. In this system, kokanee spawn on beaches near the sampled river mouths, and it remains unclear the degree to which these shore- and stream-spawning populations are reproductively isolated (Lemay and Russello 2012).

R68810 is located on linkage group So12 in Larson et al. (2017), and was the second highest FST outlier in their study, located adjacent to their highest divergence outlier (41305). This locus (Larson 41305) corresponds to our R24343, a lower differentiation outlier between shore- and stream-spawning kokanee in Okanagan lake. We mapped R68810 to the leucine-rich repeat-containing protein-9 gene (LRRC9) in both the rainbow trout (*O. mykiss* chromosome 29) and Atlantic salmon genomes (*S. salar* chromosome 9). We have further investigated the generality of this locus as an ecotype identifier across the range of *O. nerka*, as well as the surrounding genomic regions for the linked genes under divergent selection (Veale and Russello 2017).

Given the high economic and cultural value of salmonids in British Columbia, this highly informative SNP in combination with other outlier loci identified here have immediate utility for informing fisheries management throughout the province (Russello et al. 2012; Veale and Russello 2016) and likely across the entire range. Specifically, in lakes where variation at this SNP is largely fixed and different between reproductive ecotypes (such as in Okanagan Lake), relative abundances can be monitored over time using trawl or angler samples—something previously not possible with thousands of neutral markers (Lemay and Russello 2015b). This would further allow enumeration of the shore-spawning population, estimates not previously attainable as they have been for surveying stream-spawning populations through the use of fences. Furthermore, we have already shown the efficacy of the five kokanee/anadromous sockeye salmon outliers (R7641, R18232, R40949, R47083, R122749) for informing a restocking program and associated spawning habitat management, through studies of hybridization rates between kokanee and reintroduced anadromous sockeye salmon in Skaha Lake, British Columbia (Veale and Russello 2016). The resolution for describing introgression levels obtained from the five outlier loci far surpassed the signal provided by 50 putatively neutral genotyping assays (Veale and Russello 2016). Moving forward, the genomic resources generated here hold great promise for establishing SNP panels that can accurately, rapidly, and cost-effectively inform stock assessment, restocking, and other fisheries management applications at multiple spatial scales, from the lake-level to cross-drainage programs and international initiatives.

## Supplementary Material

Supplementary data are available at *Genome Biology and Evolution* online.

## Supplementary Material

Supplementary Data
