# Supplementary material for: Genomic Changes Associated with Reproductive and Migratory Ecotypes in Sockeye Salmon (Oncorhynchus nerka)
Source: Genome Biol Evol. 2017 Oct 13;9(10):2921–39. doi: 10.1093/gbe/evx215 (PMC5737441; doi:10.1093/gbe/evx215)

Figure S1. FASTSTRUCTURE (Raj et al, 2014) plot for Wood Lake kokanee based on genotypic data at 6,234 putatively neutral SNPs showing the proportion of cluster membership at K=2. Vertical bars indicate individuals, colors represent proportion of cluster membership. Colors denote reproductive ecotype (red = stream-spawning, brown = shore-spawning).

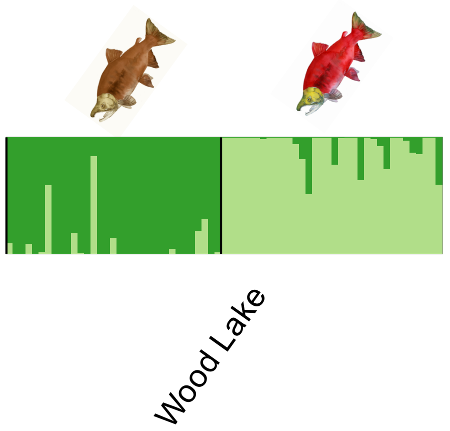

Figure S2. Principle component plots for PCs 1 – 9 as identified in PCADAPT (Luu *et al.* 2016). Due to the odd number of PCs, PC1 is displayed twice – to allow a two dimensional plot for PC9. An = Anderson Lake, Se = Seton Lake, Po = Portage Creek, KoW = Kootenay Lake West Arm, KoN = Kootenay Lake North Arm, Wo = Wood Lake, Ok = Okanagan Lake, OKR = Okanagan River, Sk = Skaha Lake, Tch = Tchesinkut Lake, K = kokanee, S = sockeye, Sh = Shore, St = Stream.

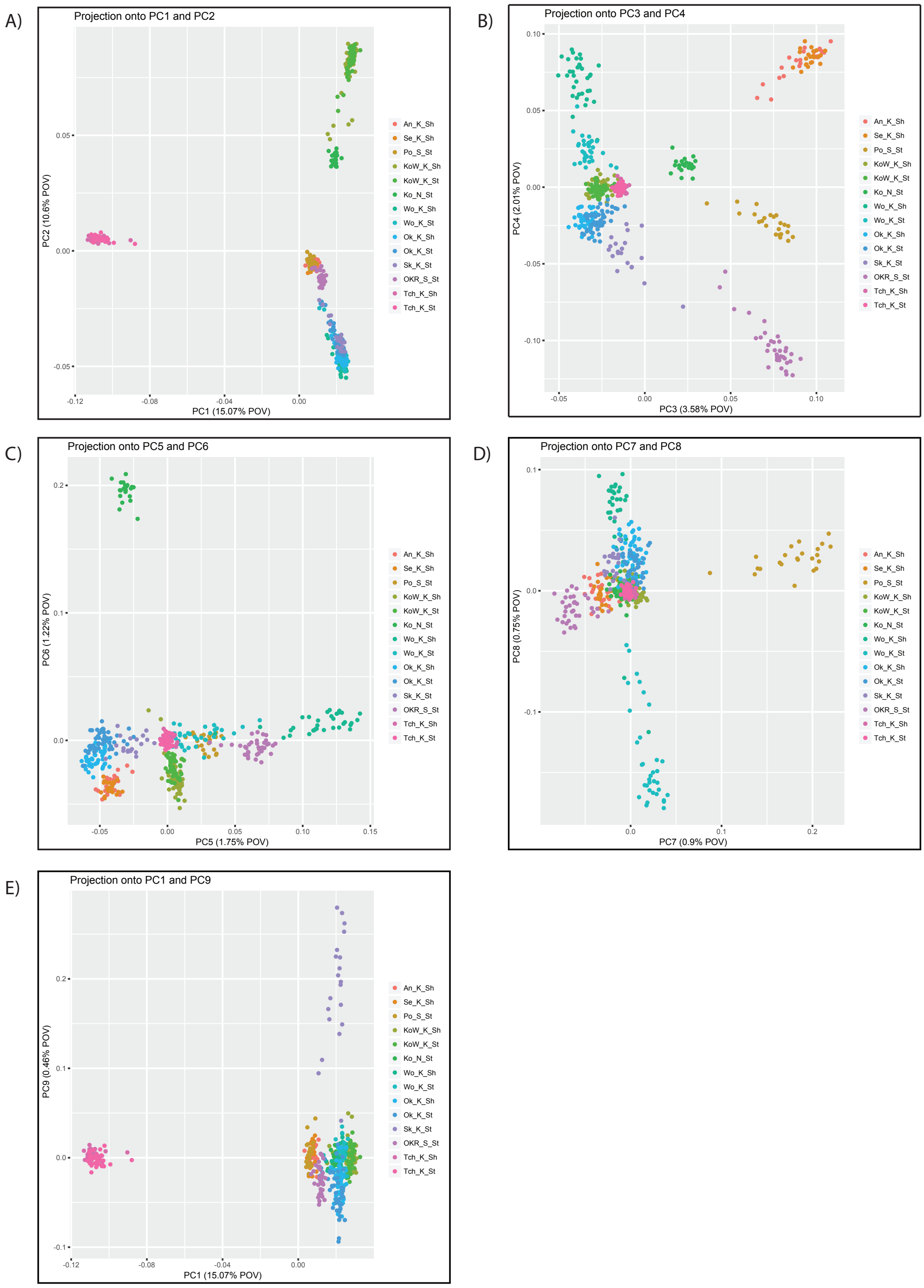

Figure S3. Principle component plots for each of the individual-lake analyses performed in PCADAPT (Luu et al. 2016). Note for panels D, E, and F only one principle component (PC1) may be significant, but the second is included to investigate further structure. An = Anderson Lake, Se = Seton Lake, Po = Portage Creek, KoW = Kootenay Lake West Arm, KoN = Kootenay Lake North Arm, Wo = Wood Lake, Ok = Okanagan Lake, OKR = Okanagan River, Sk = Skaha Lake, Tch = Tchesinkut Lake, K = kokanee, S = sockeye, Sh = Shore, St = Stream. Within Okanagan Lake sampling locations are described in Lemay & Russello (2015).

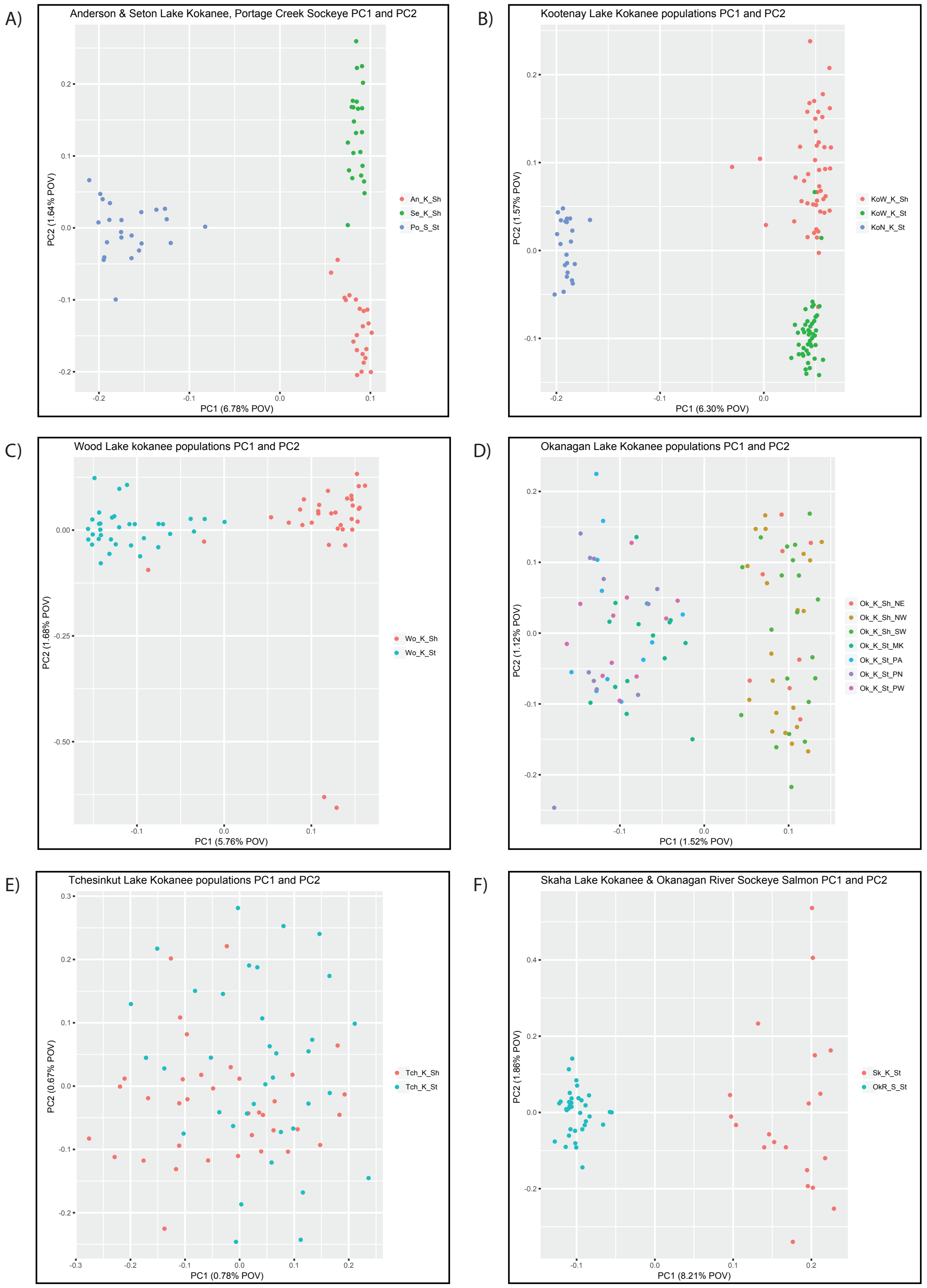

Figure S4. Plots of genetic differentiation (F_{ST}) across each linkage group in the sockeye salmon linkage map (Larson *et al*, 2016) for the three primary combined ecotype comparisons including: A) shore- versus stream-spawning kokanee; B) Anderson/Seton Lake shore-spawning kokanee versus Portage Creek anadromous sockeye; and C) Okanagan Basin lakes kokanee versus Okanagan River anadromous sockeye. Islands of divergence identified using a kernel smoothing technique are denoted as "Island regions". The "Outlier SNP" designation denotes outlier loci that are found outside of islands of divergence, and the "Island SNP" designation indicates outlier loci that are found within islands of divergence.

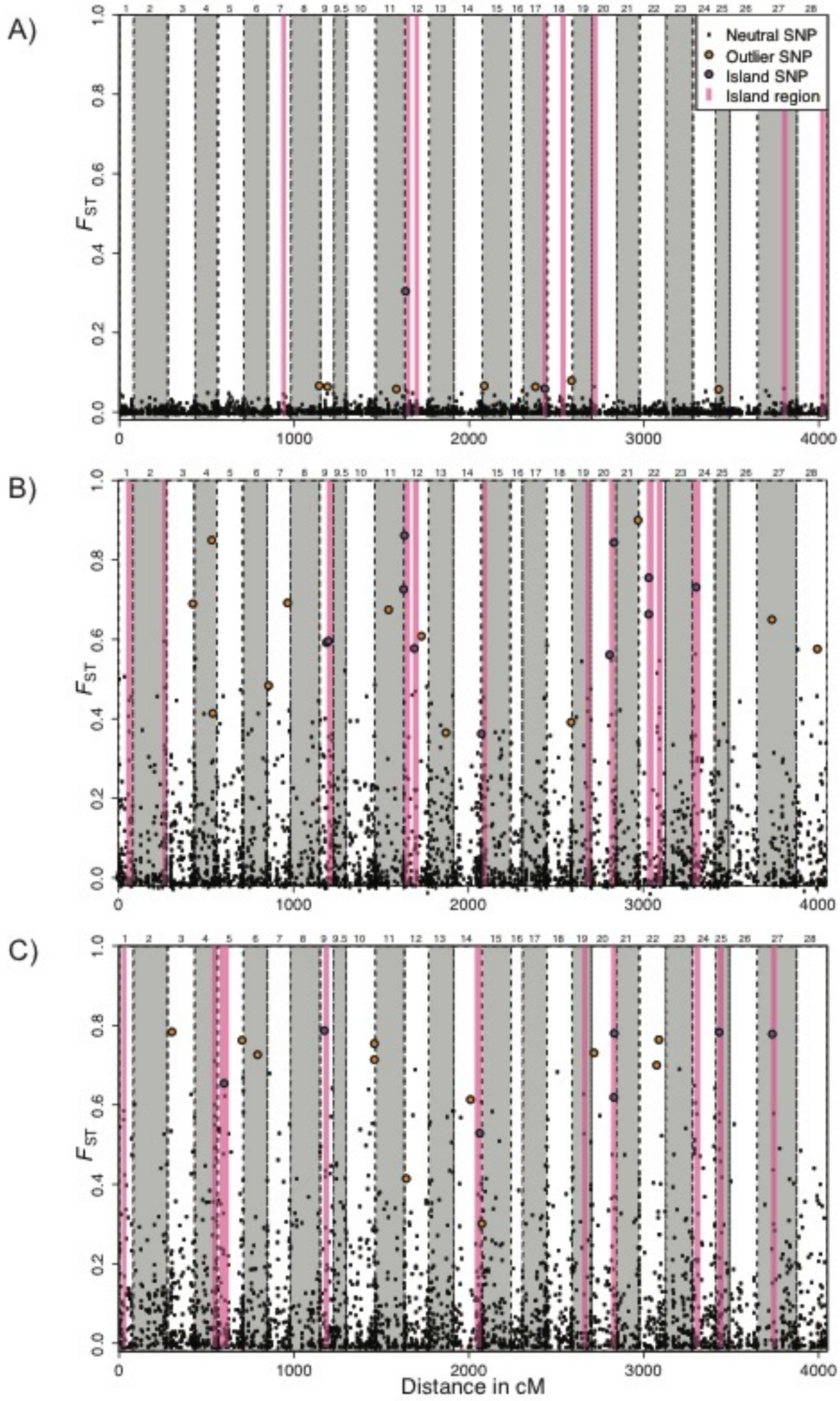

Figure S5. Heatmap of pairwise linkage disequilibrium (r^2) between outlier SNPs identified in this study. The heatmap for Okanagan Lake kokanee stream-spawners shown, which was representative across the sampling.

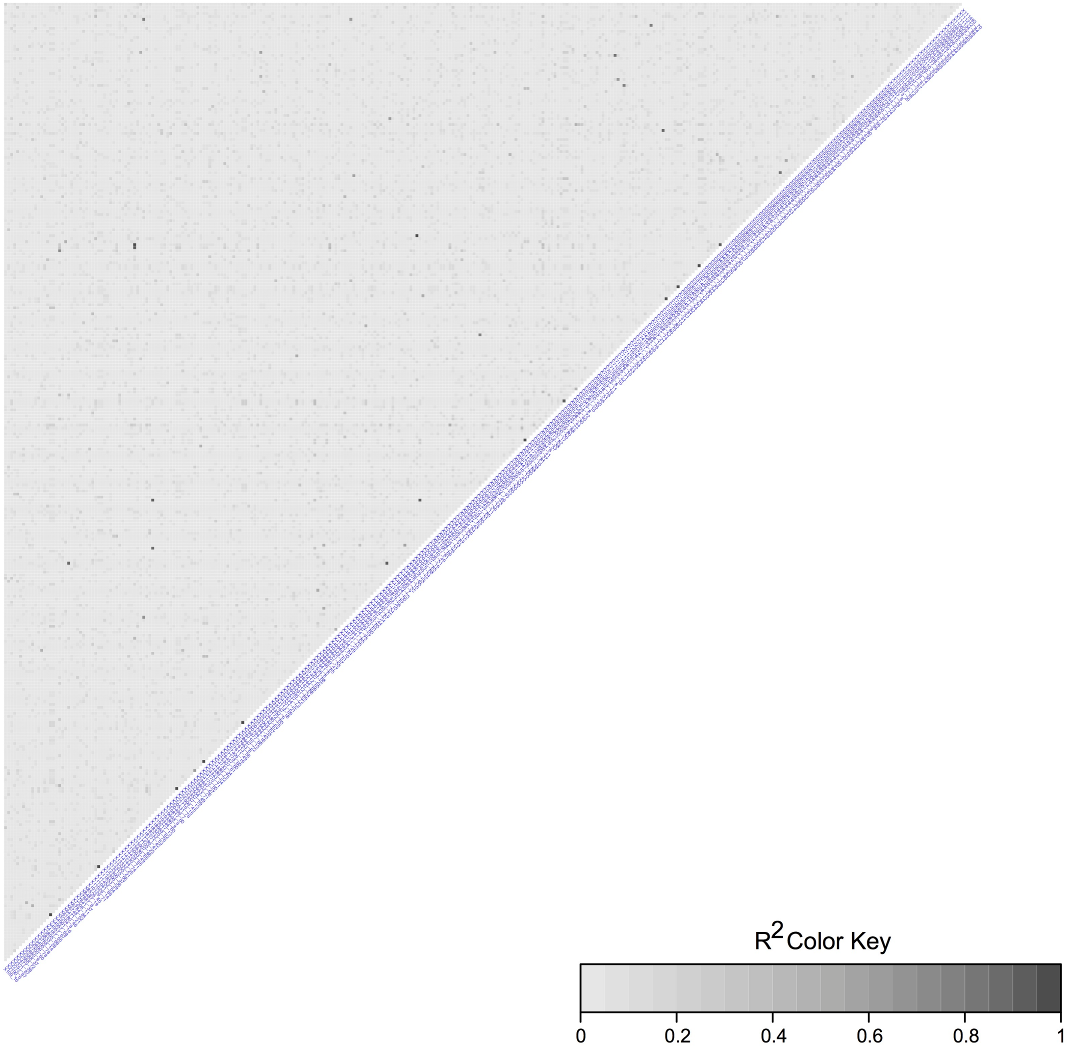

Supplement: Supplementary Data [file evx215_supp.zip › suppl_data/ESM Figures_rev.pdf]
