# Supplementary material for: Genomic Changes Associated with Reproductive and Migratory Ecotypes in Sockeye Salmon (Oncorhynchus nerka)
Source: Genome Biol Evol. 2017 Oct 13;9(10):2921–39. doi: 10.1093/gbe/evx215 (PMC5737441; doi:10.1093/gbe/evx215)
Supplement: Supplementary Data [file evx215_supp.zip › suppl_data/ESM Tables_rev.docx]

Supplementary Table 1. Weir and Cockerham's (1984) *Fst* between each sampled population.

|  | An  K_Sh | Se  K_Sh | PC  S_St | KoW  K_Sh | KoW  K_St | KoN  K_St | Wo  K_Sh | Wo  K_St | Ok  K_Sh | Ok  K_St | Sk  K_St | OkR  S_St | Tc  K_Sh |
| --- | --- | --- | --- | --- | --- | --- | --- | --- | --- | --- | --- | --- | --- |
| Se_K_Sh | 0.0103 |  |  |  |  |  |  |  |  |  |  |  |  |
| PC_S_St | 0.0882 | 0.0835 |  |  |  |  |  |  |  |  |  |  |  |
| KoW_K_Sh | 0.2158 | 0.2098 | 0.2084 |  |  |  |  |  |  |  |  |  |  |
| KoW_K_St | 0.2165 | 0.2101 | 0.2108 | 0.0100 |  |  |  |  |  |  |  |  |  |
| KoN_St | 0.1343 | 0.1296 | 0.1395 | 0.1282 | 0.1250 |  |  |  |  |  |  |  |  |
| Wo_K_Sh | 0.1680 | 0.1627 | 0.1682 | 0.2727 | 0.2740 | 0.2097 |  |  |  |  |  |  |  |
| Wo_K_St | 0.1389 | 0.1327 | 0.1394 | 0.2433 | 0.2452 | 0.1770 | 0.0591 |  |  |  |  |  |  |
| Ok_K_Sh | 0.1315 | 0.1255 | 0.1314 | 0.2322 | 0.2343 | 0.1680 | 0.0864 | 0.0446 |  |  |  |  |  |
| Ok_K_St | 0.1243 | 0.1187 | 0.1228 | 0.2225 | 0.2244 | 0.1542 | 0.0886 | 0.0440 | 0.0083 |  |  |  |  |
| Sk_K_St | 0.1261 | 0.1216 | 0.1204 | 0.2314 | 0.2335 | 0.1585 | 0.1014 | 0.0626 | 0.0316 | 0.0303 |  |  |  |
| OkR_S_St | 0.1095 | 0.1071 | 0.0867 | 0.2087 | 0.2089 | 0.1426 | 0.1561 | 0.1284 | 0.1159 | 0.1082 | 0.0918 |  |  |
| Tc_K_Sh | 0.3853 | 0.3705 | 0.3853 | 0.4522 | 0.4534 | 0.4218 | 0.4277 | 0.4069 | 0.3774 | 0.3775 | 0.4160 | 0.3782 |  |
| Tc_K_St | 0.3846 | 0.3697 | 0.3857 | 0.4524 | 0.4536 | 0.4223 | 0.4276 | 0.4066 | 0.3770 | 0.3770 | 0.4159 | 0.3779 | 0.0026 |

Population codes: KoN = Kootenay Lake North Arm, KoW = Kootenay Lake West Arm, OkR = Okanagan River, An = Anderson Lake, Se = Seton Lake, PC = Portage Creek, Sk = Skaha Lake, Ok = Okanagan Lake, Wo = Wood Lake, Tc = Tchesinkut Lake, K = Kokanee, S = Sockeye, St = Stream, Sh = Shore.

Supplementary Table 2. Outlier loci identified in multiple comparisons. Significant outliers are identified by color – orange = shore/stream comparisons, green = mixed shore/stream and kokanee/anadromous sockeye comparisons, blue = kokanee/anadromous sockeye comparisons. Columns are as follows: 1: Combined paired shore-spawning kokanee vs. stream-spawning kokanee, 2: Okanagan Basin lake kokanee vs. Okanagan River anadromous sockeye, 3: Anderson & Seton Lake kokanee vs. Portage Creek anadromous sockeye, 4: Okanagan Lake shore- vs. stream-spawning kokanee, 5: Wood Lake shore- vs. stream-spawning kokanee, 6: Kootenay Lake West Arm shore- vs. stream-spawning kokanee, 7: Tchesinkut Lake shore- vs. stream-spawning kokanee, 8: Okanagan Lake kokanee vs. Okanagan River anadromous sockeye, 9: Skaha Lake kokanee vs. Okanagan River anadromous sockeye, 10: Wood Lake shore-spawning kokanee vs. Okanagan River anadromous sockeye, 11: Wood Lake stream-spawning kokanee vs. Okanagan River anadromous sockeye, 12: Kootenay West Arm kokanee vs. Okanagan River anadromous sockeye, 13: Kootenay North Arm kokanee vs. Okanagan River anadromous sockeye, 14: Redfish Lake stream-spawning kokanee vs. Redfish Lake shore-spawning sockeye, 15: Alturas Lake kokanee vs. Alturas lake sockeye, 16: Alaskan beach- vs. stream/river-spawning sockeye. Columns 14 & 15 data from Nichols *et al.* (2016), Column 16 data from Larson *et al.* (2017). 1 = outlier, 0 = not included in study. The original RAD tag codes of Nichols *et al.* (2016) and Larson *et al.* (2017) are stated for significant shared outliers in columns 14 – 16.

Supplementary Table 3. Regions of the *Oncorhynchus nerka* linkage map (Larson et al. 2016) containing multiple outlier loci.

| Code | female LG | female pos (cM) | KoW_K_Sh/KoW_K_St | Ok_K_Sh/ Ok_K_St | Wo_K_Sh/ Wo_K_St | An_Se_K_Sh/PC_S_St | Ok_K_St_Sh/  OkR_S_St | Sk_K_St/  OkR_S_St | Wo_K_Sh/  OkR_S_St | Wo_K_St/  OkR_S_St | KoW_K_Sh/  OkR_S_St | KoN_K_St/  OkR_S_St |
| --- | --- | --- | --- | --- | --- | --- | --- | --- | --- | --- | --- | --- |
| 108326 | 2b | 167.94 |  |  |  |  |  |  |  |  | √ | √ |
| 78909 | 2b | 168.13 |  |  |  |  |  |  |  |  | √ | √ |
| 48535 | 4b | 115.71 |  |  |  |  |  |  | √ |  |  |  |
| 54026 | 4b | 115.71 |  |  |  | √ |  |  |  |  |  |  |
| 20531 | 4b | 115.71 |  |  |  |  |  | √ |  |  |  |  |
| 6784 | 11a | 3.24 |  |  |  |  | √ |  |  |  |  |  |
| 71626 | 11a | 4.19 |  |  |  |  | √ |  |  |  |  |  |
| 105110 | 12a | 6.14 |  |  |  |  |  |  |  |  | √ |  |
| 77059 | 12a | 7.79 |  |  |  | √ |  |  |  |  |  |  |
| 61735 | 12a | 7.79 | √ |  |  |  |  |  |  |  |  |  |
| 68810 | 12a | ~17 |  | √ | √ | √ |  |  | √ |  |  |  |
| 24343 | 12a | 18.34 |  | √ |  |  |  |  |  |  |  |  |
| 68420 | 13a | 19.58 |  |  |  |  |  | √ |  |  |  |  |
| 22663 | 13a | 20.27 |  |  |  |  |  |  |  |  | √ | √ |
| 93027 | 15a | 8.19 |  |  |  |  |  |  | √ | √ |  |  |
| 18325 | 15a | 8.19 |  |  |  | √ |  |  |  |  |  |  |
| 45460 | 15a | 8.19 | √ |  |  |  |  |  |  |  |  |  |
| 67017 | 15a | 12.65 |  |  |  |  |  |  |  | √ |  |  |
| 32485 | 15a | 12.65 |  |  |  | √ |  |  |  |  |  |  |
| 104237 | 18a | 5.23 |  |  |  |  |  |  |  | √ |  |  |
| 55745 | 18a | 5.26 |  |  |  |  |  |  |  | √ |  |  |
| 57502 | 20b | 140.85 |  |  |  |  | √ |  |  |  |  |  |
| 122749 | 20b | 141.26 |  |  |  | √ | √ |  | √ | √ |  | √ |
| 83558 | 22^#^ | 65.69 |  |  |  | √ |  |  |  |  |  |  |
| 101937 | 22^#^ | 65.69 |  |  |  | √ |  |  |  |  |  |  |
| 18232 | 25a | 31.52 |  |  |  |  | √ | √ | √ |  |  |  |
| 89278 | 25a | 33.42 |  |  |  |  |  |  | √ |  | √ |  |

Population codes: KoN = Kootenay Lake North Arm, KoW = Kootenay Lake West Arm, OkR = Okanagan River, An = Anderson Lake, Se = Seton Lake, PC = Portage Creek, Sk = Skaha Lake, Ok = Okanagan Lake, Wo = Wood Lake, Tc = Tchesinkut Lake, K = Kokanee, S = Sockeye, St = Stream, Sh = Shore. ^#^ = centrome.
